# Production of Chimeric Acidic α-Amylase by the Recombinant *Pichia pastoris* and Its Applications

**DOI:** 10.3389/fmicb.2017.00493

**Published:** 2017-03-22

**Authors:** Deepak Parashar, Tulasi Satyanarayana

**Affiliations:** Department of Microbiology, University of DelhiNew Delhi, India

**Keywords:** chimeric α-amylase, *Pichia pastoris*, multi-copy clones, post-transformational vector amplification, starch saccharification

## Abstract

Recombinant chimeric α-amylase (Ba-Gt-amy) has been produced extracellularly in *Pichia pastoris* under *AOX* promoter. Clones of *P. pastoris* with multiple gene copies have been generated by multiple transformations and post-transformational vector amplification, which led to 10.7-fold enhancement in α-amylase titre as compared to a clone with a copy of the gene. The recombinant *P. pastoris* integrated eight copies of *Ba-Gt-amy* in the genome of *P. pastoris*, as revealed by real time PCR data analysis. Heterologous protein expression as well as mRNA level of *Ba-Gt-amy* was higher in multi-copy clone than that with single copy. The pure Ba-Gt-amy expressed in *P. pastoris* is a glycoprotein of 75 kDa, which is optimally active at pH 4.0 and 60°C with T_1/2_ of 40 min at 70°C. The Kinetic parameters and end product analysis suggested that glycosylation has no effect on catalytic properties of Ba-Gt-amy. The enzyme saccharifies soluble as well as raw starches efficiently and generates maltose and maltooligosaccharides, thus, useful in baking and sugar syrup industries. The strategy for generating multi-copy clones is being reported for the first time, which could be useful in enhancing the production of other recombinant proteins.

## Introduction

The extracellular recombinant enzyme production simplifies recovery and downstream processing of the target protein. The methylotrophic yeast *Pichia pastoris* has emerged as an important production host for secretion of proteins for both basic research and industrial processes (Markošová et al., 2015; Spohner et al., 2015; Vici et al., 2015). Several factors have contributed to its rapid acceptance over other expression systems, including the tightly regulated strong alcohol oxidase I promotor (*AOX*), a strong respiratory growth system that facilitates high cell densities, integration of linearized foreign DNA via homologous recombination procedures to generate stable cell lines, secretion of large amounts of the target proteins and possibility of post-translational modification of the expressed proteins, such as processing of signal sequence, disulfide bridge formation and both *O*- and *N*-linked glycosylation (Cereghino and Cregg, 2000; Macauley-Patrick et al., 2005). *P. pastoris* expression system has provided a platform for commercialization of a wide range of protein products such as enzymes, antigens and antibodies. More than 70 products synthesized in *P. pastoris* are available now in the market^1^.

Several genetic and physiological factors determine the productivity of a recombinant system in *P. pastoris*. Potential bottlenecks are the codon usage of the heterologous gene (Sun et al., 2016), the gene copy number, efficient transcription and translation, processing and folding and final secretion out of the cell and protein turnover by proteolysis (Hohenblum et al., 2004; Aw and Polizzi, 2013). Among them, a commonly used approach to improve the expression level in *P. pastoris* is to increase the copy number of the expression cassette, which was shown to be effective in many cases (Clare et al., 1991; Marx et al., 2009; Nordén et al., 2011; Aw and Polizzi, 2013). Numerous examples can be cited from the literature indicating that an increase in gene copy number can significantly increase productivity provided that secretion level should not reach saturation, which suggests that there is a linear correlation between copy number and expression of heterologous proteins (Romanos et al., 1998; Vassileva et al., 2001; Aw and Polizzi, 2013).

In order to generate multi-copy integrants that can secrete high level of heterologous protein, we have used multiple transformation approach followed by post-transformational vector amplification (PTVA). Multi-copy strain generated in this investigation has proved to be useful in improving the titre of recombinant chimeric α-amylase significantly. This strategy would be useful in producing high titres of other heterologous proteins in *P. pastoris*. To the best of our knowledge, the combination of two approaches has not yet been reported.

## Materials and Methods

### Strains and Vectors

*Escherichia coli* DH5α [genotype: F*^-^ endA1 glnV44 thi-1 recA1 relA1 gyrA96 deoR nupG purB20 φ80dlac*ZΔM15 *Δ(lacZYA-argF)*U169, hsdR17$\left(r_{K}^{-}m_{K}^{+}\right)$,λ*^-^*] was used as the host strain for cloning and propagation of vector. *P. pastoris* X-33 (genotype: wild type) (Invitrogen, Carlsbad, CA, USA) was used as the expression host. *E. coli* clone harboring chimeric α-amylase gene (Ba-Gt-amy) generated earlier (Parashar and Satyanarayana, 2016b) was procured from the laboratory culture collection. pPICZαA vector (Invitrogen) was used as a cloning and expression vector. The restriction enzymes and the primers used in this investigation were purchased from New England Biolabs (Beverly, MA, USA) and Sigma-Aldrich (USA), respectively. Plasmid extraction and gel elution kits were purchased from Real Biotech Corporation (RBC) (Taiwan). Media and other components for trace metal solution used in this experiment are listed in **Table 1**.

**Table 1:** Composition of media used in this investigation.

Culture media	Composition
YPD (yeast-peptone-dextrose):gL^-1^	Yeast extract 10, peptone 20, glucose 20, and agar powder 15 (if necessary)
LSLB (low salt Luria-Bertani) gL^-1^	Yeast extract 5, NaCl 5, Tryptone 10, and agar powder 15 (if necessary)
BMGY (buffered minimal glycerol-yeast) gL^-1^	Yeast extract 10, peptone, 100 mM potassium phosphate buffer, glycerol 20 and yeast nitrogen base (1.37% W/V)
BMMY (buffered minimal methanol-yeast) gL^-1^	BMMY medium has the same composition like that of BMGY only glycerol is replaced by 1% methanol
BSM (basal salt medium) gL^-1^	Glycerol 40, K_2_SO_4_ 18.6, MgSO_4_⋅7H_2_O 14.9, KOH 4.13, CaSO_4_ 0.93, and H_3_PO_4_ 26.7 mL/L
PTM (Pichia trace metals solution 4.35 mL per liter) gL^-1^	CuSO_4_⋅5H_2_O 6, KI 0.09, MnSO_4_⋅H_2_O 3, H_3_BO_3_ 0.02, MoNa_2_O_4_⋅2H_2_O 0.24, CoCl_2_, ZnCl_2_ 20, FeSO_4_⋅H_2_O 65, Biotin 0.2, H_2_SO_4_ 5.0 mL


*pH was adjusted to 7.5 when Zeocin was used as a selection marker.*

### Culture Maintenance

*Pichia pastoris* strains were grown on YPD agar medium plates and stored at 4°C. The *E. coli* was cultured in low salt Luria-Bertani (LB) medium (pH 7.5) with appropriate concentrations of antibiotic at 37°C when needed. Cells were preserved as recommended by Invitrogen’s manual (Carlsbad, CA, USA) at -80°C.

### Construction of Vectors

**FIGURE 1:**
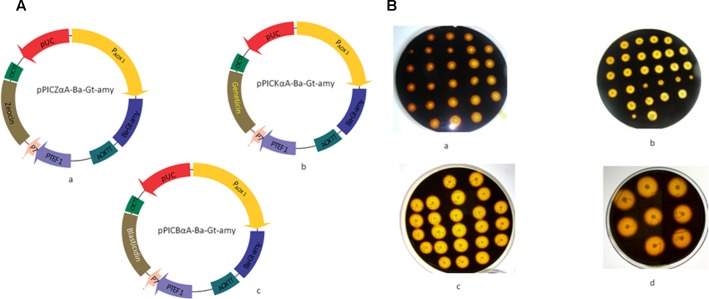
**(A)** Strategy used for the construction of expression vectors that drive the expression of α-amylase in *Pichia pastoris*. (a) pPICZαA-Ba-Gt-amy (b) pPICKαA-Ba-Gt-amy (c) and pPICBαA-Ba-Gt-amy in *P. pastoris.* **(B)** Screening of recombinant *P. pastoris* strains after each transformation. (a) Amy-AOX1 (b) Amy-AOX2 (c) Amy-AOX3 (d) Amy-AOX3 after PTVA method.

pPICZαA (Invitrogen) vector devoid of Zeocin coding region with flanking sites *Nde* I and *Nco* I sites was generated to clone kanamycin coding region as described previously (Parashar and Satyanarayana, 2016a). Geneticin (816 bp) and Blasticidin (399 bp) resistance encoding sequences were amplified from pET 28a (+) and pBC4, respectively flanking with *Nde* I and *Nco* I restriction sites and then cloned into the corresponding sites of pPICZαA to generate vector pPICKαA and pPICBαA, respectively. The pET28a-Ba-Gt-amy construct harboring the α-amylase gene was used as the template for PCR amplification of the *Ba-Gt-amy* gene by using primers P7 and P8. pPICZαA-Ba-Gt-amy, pPICKαA-Ba-Gt-amy, and pPICBαA-Ba-Gt-amy were generated by double digestion and ligation of the vectors and *Ba-Gt-amy* (**Figure 1A**). Primers used in the investigations are listed in Supplementary Table S1. All amplifications were done using Herculase II fusion DNA polymerase (Agilent) in a thermocycler (Bio-Rad, USA).

### Bioinformatic Analysis

Sim Vector 4 software was used for all vector engineering and modifications. The nucleotide and protein sequences were analyzed with the NCBI database using BLASTN and BLASTP programs, respectively. Molecular mass was predicted by using the ProtPram tool. Glycosylation sites were predicted by using the NetNGlyc 1.0 and NetOGlyc 4.0 Server.

### Transformation into *P. pastoris*

The fresh competent *P. pastoris* X-33 cells were prepared and transformed according to the Easyselect^TM^ *Pichia* expression kit manual (Invitrogen). To 800 μl of the cells, 25 μg of purified linearized plasmid DNA mixed and then pulsed in 0.1 cm electroporation cuvettes at 1.5 kV for 6 ms (Bio-Rad gene-pulser). Amy-AOX1 strain was generated by transforming wild type *P. pastoris* cells with pPICZαA-Ba-Gt-amy. To generate Amy-AOX2 strain, Amy-AOX1 strain was made competent and transformed with pPICKαA-Ba-Gt-amy. Amy-AOX2 was further made competent and transformed with pPICBαA-Ba-Gt-amy. After each transformation, clones were screened for amylase activity and clone that produced high enzyme titre was selected for further transformation.

### Post-transformational Vector Amplification with Amy-AOX3 Strain

Ten potential strains obtained after third transformation were subjected to PTVA approach as described by Sunga et al. (2008). Clone obtained after third transformation was patched onto YPD-Zeocin plates (100 μg mL^-1^ Zeocin) and left to grow for 3–4 days. Each spot was then replicated onto another plate containing increasing concentrations of Zeocin (e.g., 200, 300, 500, 1000, and 2000 μg mL^-1^ Zeocin). Clone which was capable of growth at high antibiotic levels was screened for amylase activity.

### Screening of *P. pastoris* Clones Producing Extracellular Ba-Gt-amy

Primary screening for extracellular production of Ba-Gt-amy was done by performing starch plate assay (**Figure 1B**). Clones that exhibited larger hydrolysis zones were selected for further screening in submerged fermentation.

### Determination of Gene Copy Number and Transcription Level of Ba-Gt-amy

Absolute Real time PCR quantification method was used for determining the gene copy number of the α-amylase (*Ba-Gt-amy*) in the genome of *P. pastoris*. Against *Ba-Gt-amy* region, primers P11 and P12 were designed. For constructing a standard curve, plasmid pPICZαA-Ba-Gt-amy was used as the template and against log copies of plasmid DNA, C_T_ values were plotted. The reaction mixture consisted of master mix 2x Fast SYBR green dye (final concentration 1x), forward and reverse primers (0.25 μM each), PCR grade water and template (total volume 20 μL). The thermal cycling protocol employed: initial denaturation for 10 min at 95°C followed by 40 cycles of 10 s at 95°C and annealing and extension was done at 60°C for 30 s. For conducting qPCR experiments, the Applied Biosystems 7900HT Fast Real-Time PCR System (Applied Biosystems, USA) was used. By using Biospectrophotometer (Eppendorf), the concentration of DNA in samples and corresponding DNA copies were calculated according to Lee et al. (2008). The standard curve constructed from the diluted standard template was used to determine the copy number in the genomic DNA samples by interpolation. Evaluation of the transcription of *Ba-Gt-amy* upon methanol induction was performed by quantitative real-time PCR (qPCR). Total RNA was isolated from the cultured *P. pastoris* and reverse transcribed by using reverse transcriptase PCR kit (Promega). C_T_ signals were normalized to the corresponding housekeeping gene (*GAPDH*). The data from the real-time PCR was converted to 2^-ΔΔC^_T_ [ΔΔC_T_ = (ΔΔC_T(target)_-C_T(ref)_)_1_-(ΔΔC_T(target)_-C_T(ref)_)_2_] that represents fold change (Schmittgen and Livak, 2008). All the values were analyzed through triplicate experiments. Statistical significance was considered significant at *P* < 0.05.

### Determining Stability of Integrated Plasmids in the Genome of *P. pastoris*

To determine stability of expression cassette in transformants, strains were grown on YPD medium without any antibiotic for 20 generations. Colonies were then replicated to YPD agar plates containing appropriate antibiotic and grown for 2 days. The number of viable colonies on each plate was then counted. PCR was performed from the genomic DNA of transformants to ensure integration of cassettes in the genome of *P. pastoris*.

### Recombinant Enzyme Production and Optimization in Shake Flasks

A single colony was used to inoculate 10 mL of buffered minimal glycerol-yeast (BMGY) medium in a 100 mL flask. In order to express under *AOX* promoter, the recombinant was cultivated at 30°C in an incubator shaker at 250 rpm till A_600_ of 2–6 (in about 16–18 h) was attained. The fresh (50 mL) BMGY medium was inoculated with 1% seed culture and incubated in an incubator shaker at 30°C and 250 rpm for 72 h. For assaying enzymes, aliquots were drawn at every 24 h. For induced expression under *AOX* promoter, cells were harvested from 24 h old primary culture by centrifuging at 1500–3000 × *g* for 5 min and resuspended in BMMY medium till A_600_ reached 1.0. Methanol (100%) was added to a final concentration of 0.5% at every 24 h interval to maintain induction, and samples were drawn to analyze the expression levels. For optimum temperature determination, recombinant strains were grown at different incubation temperatures (16, 20, 25, and 30°C), and for determining optimum pH for production, different buffered media of varied pH (3–8) were used.

### Methanol and Glycerol Analysis

Using gas chromatograph (Shimadzu, Japan) equipped with a Restec Rtx-5 column (30 m × 0.53 mm ID × 0.25 μm df), an automatic injector and a FID detector, methanol concentration was determined. Nitrogen and hydrogen were used as carrier and as fuel gas, respectively. Injector temperature was 200°C and that of the detector was 280°C. The temperature of the oven was maintained at 40°C for 2 min, followed by ramping at 20°C min^-1^ to attain 200°C, which was maintained at this temperature for 5 min. The internal standard used was methanol. Glycerol assay kit (Sigma, USA) was used for determining glycerol.

### Amylase Assay

The activity of α-amylase was determined according to Parashar and Satyanarayana (2016b). The reaction mixture consisted of 0.5 mL of 0.5% potato starch (Sigma) prepared in 100 mM sodium acetate buffer (pH 4.0) and 0.5 mL appropriately diluted enzyme (0.21 U), incubated for 15 min at 60°C. The 3, 5- dinitrosalicylic acid (DNSA) reagent (Miller, 1959) was used to quantitate reducing sugars liberated from starch by enzyme action. One unit of α-amylase has been defined as the quantity of enzyme that releases 1 μmol of reducing sugar as maltose min^-1^ under the assay conditions. The protein concentration was determined as described by Bradford (1976) using bovine serum albumin as the standard.

### Oxygen Mass Transfer Coefficient (K_L_a), Cell Biomass and Specific Growth Rate Determination

Dynamic gassing out method was used for determining K_L_a. The aeration and agitation were stopped till dissolved oxygen (DO) dropped to critical lower limit (20%), thereafter agitation and oxygen supply resumed again and change in DO concentration was monitored. The values of K_L_a have been calculated from the rate of change of the DO with time during reoxygenation stage (Parashar and Satyanarayana, 2016a). Cell density was expressed as dry cell weight (dcw), which was estimated by centrifuging the samples at 5000 × *g* for 10 min, followed by washing the pellet twice with distilled water and drying at 80°C to a constant weight.

The specific growth rate (μ) for a fed batch culture is expressed as:

μ=d(XV)/(XV)dt

where cell density, culture volume, and time are represented as X, V, and t, respectively, and from the slope of ln(XV) versus t, μ was determined. At each sampling point, t and V were determined from the sum of the volumes of initial medium, liquid ammonia, glycerol, and methanol by subtracting the volume sampled.

### High Cell Density Fed Batch Cultivation

Experiments were conducted in a 7 L glass autoclavable fermenter (Applikon Biotech, Ltd, Netherlands) with the initial working volume of 2 L. 50 ml YPD medium was inoculated with a single colony of Amy-AOX3 and cultivated for 20–24 h at 30°C and 250 rpm till A_600_ of 6–8 was attained. The pH of the fermentation basal salts medium was adjusted to 5.5 using conc. aqueous ammonia, after sterilization. Four mL of *Pichia* Trace Metal solution (PTM) per liter of fermentation basal salts medium was added aseptically. Thereafter, fermenter was inoculated from the seed culture and cells were allowed to grow in batch mode until all the glycerol was exhausted as indicated by sharp DO spike (in about 20–24 h). After the batch mode, fed batch with 50% (w/v) glycerol feed containing 12 mL PTM per liter was started at the constant feed rate of 12 gL^-1^ h^-1^ of initial fermentation volume for about 10–12 h until a cell yield of ∼65 DCW gL^-1^ (∼220 WCW gL^-1^) was attained. Thereafter, methanol feed was started at 2.5 gL^-1^ h^-1^; after 4 h, feed was increased gradually up to 9 gL^-1^ h^-1^ and maintained throughout the subsequent fermentation. Throughout the fermentation, DO level was maintained above 35% by adjusting agitation rate (500–1100 rpm) and supplying air (1–2 vvm) or pure oxygen (0.1–0.3 vvm). To analyze cell growth, microscopic purity and enzyme activity, fermented medium samples were collected at 12 h intervals.

### Purification and Analysis of SDS-PAGE, Glycosylation, and Zymogram

Cell free supernatant was first passed through ultrafiltration using 10 kDa ultrafiltration membrane cartridge (Millipore), followed by fractional acetone precipitation (30–50%). The purification of the enzyme to homogeneity was carried out by anion exchange chromatography using Q-sepharose as anion exchanger. The protein samples were analyzed using 10% SDS-PAGE under reduced conditions and the gel was stained with Coomassie Brilliant blue dye. The zymogram analysis was performed by loading the purified enzyme on 10% SDS-PAGE containing 0.1% starch.

### Biochemical Characterization and Enzyme Kinetics of Ba-Gt-amy Synthesized by *P. pastoris*

The optimum pH for the activity of Ba-amy was determined by performing enzyme assays at varied pH [100 mM sodium acetate buffer (pH 3–4), 100 mM potassium phosphate buffer (pH 5–8), and glycine-NaOH buffer (pH 9–11)] at 60°C. Amylase assays were performed at varied temperatures (40–90°C) to determine optimum temperature. The pH and temperature stability were determined by incubating the enzyme at varied pH, and at various temperatures for different time intervals followed by determining residual activities. The effect of various modulators on Ba-Gt-amy activity was assessed by performing enzyme assays in their presence. The K_m_, V_max_, and K_cat_ values were graphically determined from the Lineweaver–Burk plot.

### Substrate Spectrum and Applicability of Glycosylated Ba-Gt-amy in Saccharification of Raw Starches

Substrate spectrum of Ba-Gt-amy was analyzed by conducting enzyme assays with different substrates (soluble starch, wheat flour, corn starch, water chestnut, rice flour, α- and β-cyclodextrins, amylose, and amylopectin). Raw starch saccharification was analyzed by dispensing different raw starches (15% w/v) into 100 mL flasks, gelatinized at 100°C followed by hydrolysis with the Ba-Gt-amy (10 U g^-1^ starch). Aliquots were drawn at the desired intervals for analyzing the end products. The saccharification reaction was terminated after 24 h and the extent of starch hydrolysis was calculated as follows:

$$\%\text{Starch saccharification}=\frac{\text{Reducing sugars}\;(\mu \text{g/mL})\text{as maltose}\times 0.95\times 100}{\text{Initial weight of starch }\left(\text{mg/mL}\right)}$$

The factor 0.95 normalizes the conversion for the weight gain caused by the addition of water molecule to glycosyl moiety on hydrolysis.

### Analysis of Sugars

The end products were analyzed by TLC as described by (REF). The products were further confirmed by HPLC (Shimadzu) equipped with RID detector using Aminex HPX-87H column (Bio-Rad, USA). The mobile phase consisted of acetonitrile (HPLC grade) and water (70:30 v/v). Elution was carried out at 0.5 mL/min flow rate and room temperature.

## Results and Discussion

### Cloning and Expression of *Ba-Gt-amy* in *P. pastoris*

In our previous report (Parashar and Satyanarayana, 2016b), we constructed a chimeric amylase (Ba-Gt-amy) having catalytic domain from acidic amylase of *B. acidicola* and N- and C- terminal additional amino acids from thermophilic α-amylase of *Geobacillus thermoleovorans*. This chimeric amylase has been successfully expressed in *E. coli* and shown to be useful in the saccharification of raw starches at its native pH of 3.0–5.0 without Ca^2+^ (Parashar and Satyanarayana, 2016b). Despite high titre of Ba-Gt-amy in *E. coli*, intracellular expression is a major bottleneck because its recovery needs energy intensive cell disruption methods. In order to attain extracellular expression of Ba-Gt-amy, it was cloned and expressed in *P. pastoris* under *AOX* promoter. Codon analysis of *Ba-Gt-amy* revealed the feasibility of its expression in *P. pastoris* (Supplementary Table S2). The expression vector pPICZαA gets integrated into the genome of *P. pastoris* via homologous recombination. Therefore, theoretically the copy number of the expression cassettes integrated into the *P. pastoris* determines the expression level of heterologous protein, because increasing the gene dosage will increase the specific transcript and the increased transcript level leads to an increase in specific translation. In order to elucidate this, we generated three constructs of pPICZαA with three different resistance markers (pPICZαA, pPICKαA, and pPICBαA vectors) (**Figure 1A**). pPICKαA vector confers resistance against kanamycin in bacteria and Geneticin (G-418) in yeast. The pPICBαA confers resistance against Blasticidin in both bacteria and yeast.

Three *P. pastoris* strains (Amy-AOX1, Amy-AOX2, and Amy-AOX3), which contain Ba-Gt-amy were generated. Amy-AOX1 was generated by transforming with pPICZαA-Ba-Gt-amy. Amy-AOX1 was made further competent and transformed with pPICBαA-Ba-Gt-amy to generate Amy-AOX2. Similarly, Amy-AOX2 was made competent and transformed with pPICBαA-Ba-Gt-amy. After third transformation, a total of 10 strains that produced higher titre of Ba-Gt-amy than Amy-AOX2 were selected and subjected to PTVA. Among potential strains, a strain was selected for further investigation and designated as Amy-AOX3. This strain showed resistance to Zeocin, G-418 and Blasticidin, and thus, confirmed successful integration of all three plasmids. Further, stability of integrated plasmid was checked by growing recombinant cells YPD medium without any antibiotic for 20 generations. Colonies were then replicated on YPD agar plates containing appropriate antibiotic and grown for 2 days. The number of viable colonies on each plate was then counted. PCR was also performed from the genomic DNA of transformants to ensure integration of cassettes in the genome of *P. pastoris*. A total of threefold improvement in Ba-Gt-amy titre was attained over that of Amy-AOX1 strain (**Table 2**). Multiple transformations by using different selection markers have an advantage that each transformation allows generation of clones with higher copy number as compared to that of previous one. In earlier reports, it has been reported that increase in copy number can lead to enhancement in intracellular protein titres (Clare et al., 1991; Vassileva et al., 2001; Marx et al., 2009). In this investigation, we are reporting increase in the titre of secretory protein with increase in gene copy number. Production was further enhanced by using PTVA approach. A 1.25-fold improvement in amylase production was attained with the PTVA approach. PTVA method has been shown to be useful in enhancing the titre of proteins (Sunga et al., 2008; Marx et al., 2009; Aw and Polizzi, 2016). The increase in production because of PTVA method could be due to second round of integration with more free plasmids in the chromosome of *P. pastoris*, when the strain was exposed gradually to the increased concentrations of the antibiotic (Aw and Polizzi, 2016).

**Table 2:** Improvement in the production of Ba-Gt-amy in *Pichia pastoris.*

Strain	Amy-AOX1	Amy-AOX2	Amy-AOX3^∗^	Amy-AOX3^∗∗^	After optimization	Fed batch fermentation
Enzyme production (U/mL)	70 ± 2	150 ± 2.2	200 ± 2.5	250 ± 3	310 ± 3.1	750 ± 4.5
Fold improvement	1	2.1	2.9	3.6	4.3	10.7
Cell biomass (dcw) g/L	14 ± 0.2	13 ± 0.1	13 ± 0.3	13 ± 0.2	14 ± 0.1	80 ± 1.5


*Results are an average of three separate experiments, SD within 5%. ^∗^Generated from Amy-AOX2 by transformation. ^∗∗^Generated from Amy-AOX3 by PTVA method.*

Real time PCR confirmed that Amy-AOX1, Amy-AOX2, and Amy-AOX3 contained 1, 3, and 5 copies of chimeric α-amylase gene, respectively. After PTVA, Amy-AOX3 showed the presence of eight copies of chimeric α-amylase gene. The gene copy number showed correlation between the recombinant protein expression level and the number of inserted gene copies of *Ba-Gt-amy*, since production levels increased with increase in gene copy number (**Figure 1B**). In order to study the change in the gene (*Ba-Gt-amy*) expression level due to increase in copy numbers, we performed relative qPCR analysis of all *P. pastoris* clones. Transcription of the *Ba-Gt-amy* upon methanol induction was found to be fivefold higher than Amy-AOX1 (**Table 3**). There was no decrease in amylase production due to repeated transformation, suggested that total gene copy number was lower than the threshold value. Gene copy number beyond a threshold level has been reported to lower the titre of heterologous proteins (Hohenblum et al., 2004; Love et al., 2012). This threshold value will depend on the product as well as on the specific properties of the expression construct. The most widely accepted explanation for the limited gene dosage is that the depletion of precursors and energy which might become limited at a certain level of gene copy number due to exhaustion of cell machinery (Hohenblum et al., 2004; Zhu et al., 2009; Aw and Polizzi, 2013).

**Table 3:** Expression profile of *Ba-Gt-amy* from three different strains determined using real time RT-PCR.

Culture	Amy-AOX1	Amy-AOX2	Amy-AOX3	Amy-AOX3^∗^
C_T_ GAPDH (reference)	19.0 ± 0.2	19.2 ± 0.1	19.1 ± 0.1	19.0 ± 0.08
C_T_ Ba-Gt-amy (target)	18.3 ± 0.1	17.7 ± 0.2	16.4 ± 0.2	16.05 ± 0.01
Fold change	1	1.7	4	4.7
*Ba-Gt-amy* copy number	1	3	5	8


*^∗^Amy-AOX3 after PTVA method. All the values were analyzed through triplicate experiments with ± indicating standard deviation (SD) about the mean of the triplicates. Statistical significance was considered significant at *P* < 0.05.*

Growth of strain on the medium devoid of the antibiotic did not trigger loss in any of the marker genes, suggested that the resistance marker was stably integrated into the cells. The stability was further validated by performing PCR of the genomic DNA of transformants using gene specific primers.

### Optimization of Ba-Gt-amy Production by *P. pastoris*

**FIGURE 2:**
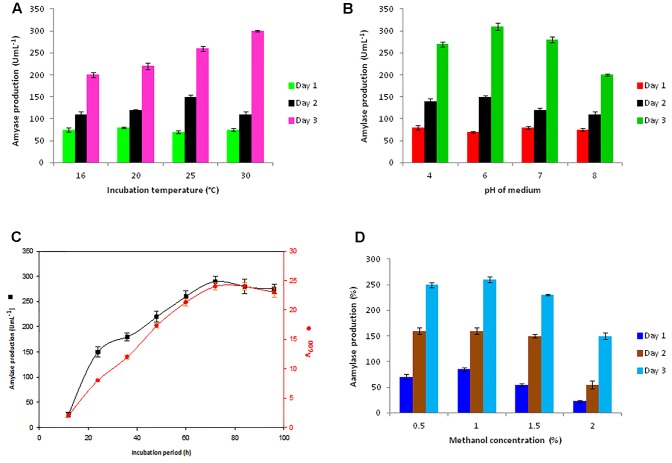
**Optimization of parameters for extracellular production of Ba-Gt-amy in *P. pastoris.*** **(A)** optimization of temperature, **(B)** pH of medium, **(C)** incubation period, and **(D)** methanol concentration for induction. [The data are an average of three separate experiments ± indicates standard deviation (SD) about the mean of the triplicates. Comparative analysis of data of all strains was done through *t*-test, and *P*-values were less than < 0.0003.]

For getting a high expression level of the Ba-Gt-amy, some key variables of the cultivation (initial pH of medium, incubation temperature, and methanol concentration) were optimized for Amy-AOX3 strain (**Figure 2**). From the growth profile, it was observed that the change in pH or temperature from optimum values had a negative effect on both cell growth and Ba-Gt-amy production, as reported earlier by Várnai et al. (2014) that optimum cultivation conditions favor recombinant protein expression. In contrast, increase in the yield of heterologous protein on altering temperature or pH beyond the optimum was observed; this could be because it facilitates folding and assembly of heterologous protein due to decrease in the protease activity of *P. pastoris* (Dragosits et al., 2009; Maurya et al., 2017).

A high enzyme titre (310 U mL^-1^) was attained from Amy-AOX3 strain in 72 h with 1% methanol as a sole carbon source (**Figure 2**). The analysis of the localization of Ba-Gt-amy in *P. pastoris* revealed that ∼98% Ba-Gt-amy produced by *P. pastoris* was extracellular with almost negligible membrane bound or intracellular activity. After optimization, a 4.3-fold increase in the production of Ba-Gt-amy was attained under AOX promoter. The growth of Amy-AOX3 was marginally reduced as compared to the wild type strain; this could be due to the metabolic burden on the cells. The toxic effect of Ba-Gt-amy expression on the growth rate of cells was not observed, contrary to that reported by Cos et al. (2005).

### Ba-Gt-amy Production by *P. pastoris* in 7 L Laboratory Fermenter

Parameters optimized in the shake flasks were replicated in the laboratory fermenter. High cell density fed batch fermentation was carried out with AOX-Amy3 strain in 7 L bioreactor using a synthetic medium. Oxygen mass transfer coefficient (K_L_a), which is a crucial parameter in high cell density fermentation, was determined; this was found to be adequate for this fermentation. The K_L_a is used as a measure of aeration capacity of a fermenter. A larger K_L_a value signifies higher aeration capacity of the system (Zhang et al., 2005). K_L_a values increased at the start of the fermentation during initial 60–72 h (Supplementary Table S3). Since temperature was kept constant throughout the cultivation, rise in K_L_a value could be attributed to increase in agitation rate and supply of air/oxygen for maintaining DO within the permissible limits. Agitation and aeration affect ‘K_L_’ and ‘a’ by reducing the resistance and changing the number or size of the air bubbles (Zhang et al., 2005). However, in the last stage of fermentation, K_L_a values declined, which might be due to the rheological properties of the cultivation medium. At high cell density, cells coalescence at high concentration of metabolites and proteins due to secretion or lysis of dead cells in the medium that leads to increase in resistance for oxygen mass transfer (Celik et al., 2009). The K_L_a values recorded in this investigation were in the higher side (0.029–0.147) than the previously reported values (0.01–0.06) (Celik et al., 2009); this could be due to the dimensions of the fermenter used.

**FIGURE 3:**
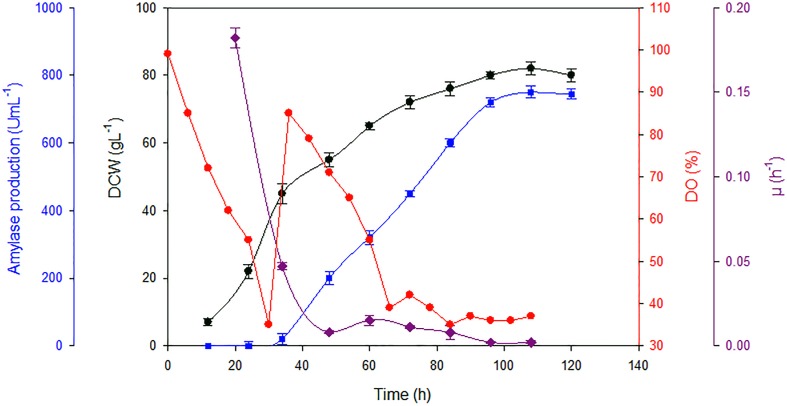
**Biomass, specific growth rate and amylase activities attained in fed batch fermentation with Amy-AOX3 strain.** [DCW (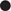); amylase activity (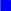); specific growth rate (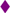) and DO (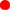)] [Data are an average of three separate experiments ± indicates standard deviation (SD) about the mean of the triplicates.]

In high cell density fermentation, approximately 2.4-fold higher titre was attained than that in the shake flasks (**Table 2**). The increase in the titre of Ba-Gt-amy is due to high biomass attained in the fermenter because of the ease in the maintenance of growth parameters (**Figure 3**). Specific activity of α-amylase was 1180 U mg^-1^. A total of 120 mgL^-1^ of Ba-Gt-amy was produced that corresponds to around 16% of total secreted soluble protein by Amy-AOX3 strain.

### SDS-PAGE Analysis of the Purified Ba-Gt-amy

**FIGURE 4:**
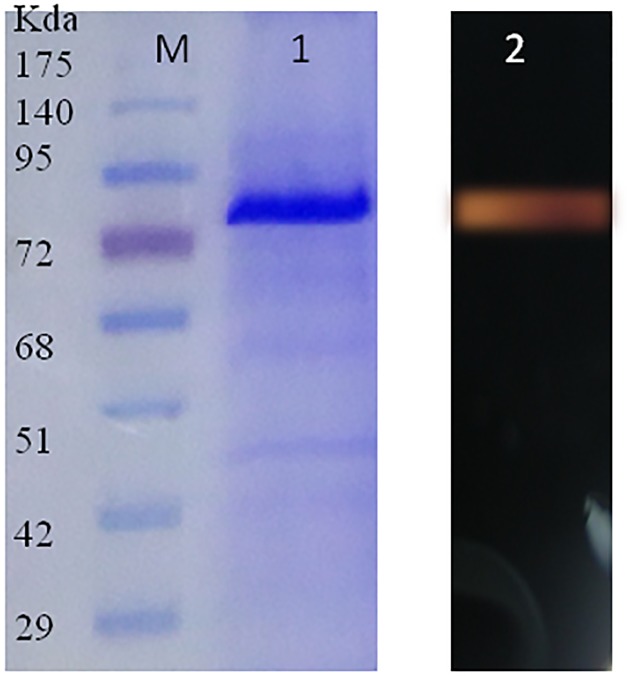
**SDS-PAGE profile of purified Ba-Gt-amy.** [M-protein marker; 1-recombinant Ba-Gt-amy purified from *P. pastoris*, 2-zymogram of Ba-Gt-amy.]

The SDS-PAGE profile of Ba-Gt-amy purification is shown in **Figure 4**. The purity of chimera was confirmed by SDS-PAGE and zymogram, and the bands correspond to approximately 68 kDa, which is slightly higher than the molecular mass of Ba-Gt-amy expressed in *E. coli*. This is due to glycosylation of protein; Ba-Gt-amy has 6 *N*- and 6 *O*-glycosylation sites as identified by the online server (Supplementary Figure S1). Glycosylation of proteins is known to increase the molecular mass of the proteins (Kumar and Satyanarayana, 2015).

### Biochemical Characterization of Ba-Gt-amy

**FIGURE 5:**
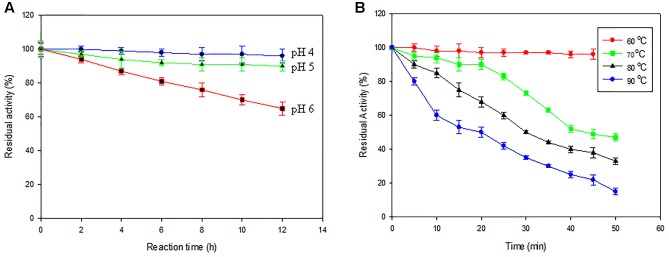
**Biochemical properties of enzymes.** **(A)** Effect of pH [pH 4 (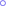); pH5 (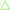); pH6 (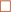] and temperature on enzyme activity of Ba-amy. **(B)** [60°C (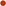); 70°C (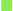); 80°C (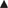); 90°C (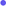)] The activity of the enzyme (0.015 mg mL^-1^) of 0 h was taken as 100 [Data are an average of three separate experiments ± indicates standard deviation (SD) about the mean of the triplicates].

The pH profile of Ba-Gt-amy expressed in *P. pastoris* resembles that of Ba-Gt-amy of *E. coli*. Ba-Gt-amy is active in the pH range between 3.0 and 6.0 with optimum at 4.0 (Supplementary Figure S2, **Figure 5**). There was no change in temperature profile of Ba-Gt-amy. The optimum temperature (60°C) and T_1/2_ of 40 min at 70°C (Supplementary Figure S2, **Figure 5**) were also similar to that expressed in *E. coli*. Thus it was concluded that the glycosylation of protein did not affect pH stability and thermostability of Ba-Gt-amy. On the contrary, Kumar and Satyanarayana (2015) reported an increase in thermostability of glycosylated xylanase expressed heterologously in *P. pastoris.* Kinetic parameters (K_m_ = 0.79 mg mL^-1^, V_max_ = 10623 μmol mg^-1^ min^-1^, and *K*_cat_ = 4 × 10^4^ s^-1^) of Ba-Gt-amy expressed in *P. pastoris* were also found to be similar to those of the recombinant Ba-Gt-Amy expressed in *E. coli*.

### Effect of Various Additives on Ba-Gt-amy

The behavior of the recombinant enzyme expressed in *P. pastoris* in the presence modulators was similar to that of Ba-Gt-amy expressed in *E. coli* (Supplementary Table S4). The amylase activity was not affected by EGTA, Ca^2+^, Na^+^, and K^+^, while the presence of Co^2+^ and Mg^2+^ stimulated the activity of Ba-Gt-amy. The cations Mn^2+^, Cu^2+^, Pb^2+^ and Ni^2+^ inhibited the activity, while in presence of Hg^2+^, Ba-Gt-amy loses activity significantly. A strong inhibition of Ba-amy by Hg^2+^ indicated oxidation of indole ring and interaction with aromatic ring of tryptophan (Dey et al., 2002). The lack of any change in Ba-amy activity in the presence of Ca^2+^ and EGTA confirmed that glycosylated Ba-Gt-amy is Ca^2+^-independent as reported earlier (Parashar and Satyanarayana, 2016b). Ba-Gt-amy inhibited activity in presence of Woodward’s reagent suggested that catalytic role of aspartic and glutamic acid residues. Woodward’s reagent K is a negatively charged reagent that covalently and irreversibly modifies carboxyl-containing amino acid residues (Adsul et al., 2009; Kumar and Satyanarayana, 2013). Strong inhibitory effect of NBS indicated structural and catalytic role of tryptophan residues in Ba-Gt-amy (Sharma and Satyanarayana, 2012).

### Substrate Spectrum and Raw Starch Saccharification

Ba-amy acts on a wide array of substrates, but not on cyclodextrins. The hydrolysis of raw starches revealed that Ba-Gt-amy saccharifies raw starches to a varied extent. Ba-Gt-amy saccharifies raw wheat (32 ± 2.12%) buck wheat (28.1 ± 2.21%), and corn (28 ± 1.5%) starches more efficiently than other starches. Sugar yield increases with increase in the concentration of enzyme and incubation period, thereafter reaches a plateau. Maximum saccharification occurs in 16 h with 20 U of Ba-Gt-amy per mg of starch (Supplementary Table S5). The rate of hydrolysis of different starches varies because digestion of raw starches by α-amylase depends on various factors like granule size, ratio of amylose to amylopectin and lipid content (Mehta and Satyanarayana, 2013). Owing to its ability to liberate maltose, maltotriose and other malto-oligosaccharides from starch at acidic pH, the enzyme can be used for simultaneous starch liquefaction and saccharification along with glucoamylase (Supplementary Figure S3) (Nisha and Satyanarayana, 2013). The acidstable, thermostable and Ca^2+^ -independent amylases reduce the cost and time required for multistep MOS production from raw starches (Sharma et al., 2016). To date, several amylases have been reported with optimum pH activity at 4–7 (Khemakhem et al., 2009; Asoodeh et al., 2010; Sharma and Satyanarayana, 2013; Ozturk et al., 2014). However, the acid stability, Ca^2+^ independence, thermostability, raw starch saccharification and a very high K_cat_ value of Ba-Gt-amy make it distinct from others.

## Conclusion

The chimeric α-amylase producing recombinant *P. pastoris* was generated through multiple transformations and selection followed by PTVA approach. This investigation suggests the possibility of enhancing extracellular recombinant protein titres by integrating increased number of gene copies into the genome of *P. pastoris*. The glycosylated recombinant chimeric α-amylase is useful in starch saccharification that liberates maltose, maltotriose and other higher malto-oligosaccharides as the major hydrolysis products.

## Author Contributions

DP and TS equally contributed in conducting experiments, analyzing results and writing the manuscript.

## Conflict of Interest Statement

The authors declare that the research was conducted in the absence of any commercial or financial relationships that could be construed as a potential conflict of interest.

## Footnotes

1 http://www.pichia.com
