# Supplementary material for: Production of Chimeric Acidic α-Amylase by the Recombinant Pichia pastoris and Its Applications
Source: Front Microbiol. 2017 Mar 22;8:493. doi: 10.3389/fmicb.2017.00493 (PMC5360700; doi:10.3389/fmicb.2017.00493)
Supplement: Supplementary file 1 [file Table_1.docx]

**Production of chimeric acidic α-amylase by the recombinant *Pichia pastoris* and its applications**

**Deepak Parashar and T. Satyanarayana***

*Department of Microbiology, University of Delhi South Campus, New Delhi-110021, India

**Corresponding author:**

Prof. T. Satyanarayana

Department of Microbiology

University of Delhi South Campus

Benito Juarez Road, New Delhi-110 021, India

Tel.: +91-11-25000132;

Fax: +91-11-24110876

E-mail addresses: [tsnarayana@gmail.com](mailto:tsnarayana@gmail.com) (T. Satyanarayana)

[dparashar2@gmail.com](mailto:dparashar2@gmail.com) (Deepak Parashar)

**Supplementary Figures**

**Fig. S1** Predicted N-glycosylated sites of Ba-Gt-amy identified by using the online NetNGlyc 1.0 server

**Fig. S2** (a) Influence of temperature (b) and pH on the activity of Ba-Gt-amy. The activity of the enzyme (0.01 mg mL^-1^) of 0 h was considered as 100 [Data are an average of three separate experiments. ± indicates standard deviation (SD) about the mean of the triplicates]

**Fig. S3** Analysis of hydrolysis products of raw starch by glycosylated Ba-Gt-amy. M-standrd markers; lane 1,2 and 3- products generated at different time intervals.

**Table S1.** Primers used in this investigation

| Primers | Products | Oligonucleotide Sequence |
| --- | --- | --- |
| P1 | *GAPDH qPCR* | AACGAAATCACCGTTTTCCA |
| P2 | *GAPDH qPCR* | AGTGTATTTCTCCTCGTTGA |
| P3 | *BlastF* | TACGACCATGGATGGCCAAGCCTTTGTC |
| P4 | *BlastR* | GGTCACATATGTTAGCCCTCCCACAC |
| P5 | *KanF* | CTAAACCATGGGCCATATTCAACGGG |
| P6 | *KanR* | TTCCACATATGTTAGAAAAACTCATCGAG |
| P7 | *Ba-Gt-amyF* | CACCGCGGCCGCTTTAACGGCACCATGATGCAGTG3’ |
| P8 | *Ba-Gt-amyR* | AACCGAA TCTAGAAGGCCATGCCACCAACCG3’ |
| P9 | *pPICZαA vector devoid of Zeocin region* | TCTACCATGGTTTAGTTCCTCACCTTGTCGTA |
| P10 | *pPICZαA vector devoid of Zeocin region* | AACGCATATGACGTCCGACGGCGGCCCACGGGT |
| P11 | *Ba-Gt-amy*  *qPCR* | GGACGAAAGCCGAAAATTG |
| P12 | *Ba-Gt-amy*  *qPCR* | TTCAGCTCGGTCACGACT |

**Table S2.** Codon analysis of *Ba-Gt-amy* gene for expression in *P. pastoris.*

|  | Actual Value | Ideal Value | Explanation |
| --- | --- | --- | --- |
| CAI* | 0.63 | > 0.5 | A CAI of 1.0 is considered ideal. The lower CAI indicates, that gene will be expressed poorly. |
| GC Content | 50.47% | 30%-70% | The ideal percentage range of GC content is between 30% and 70%. Any peaks outside of this range will adversely affect transcriptional and translational efficiency |
| CFD** | 11% | <30% | Percentage of rare codons present in BA-Gt-amy that can reduce the efficiency of translation. |

^*CAI-Codon Adaptation Index^

^**CFD-Codon distribution frequency^

**Table S3** Volumetric mass transfer coefficient K_L_a determined in high cell density fermentation

| Time  (h) | Amy-AOX3  K_L_a (S^-1^) |
| --- | --- |
| 12 | 0.027 |
| 24 | 0.077 |
| 36 | 0.113 |
| 40 | 0.12 |
| 60 | 0.135 |
| 72 | 0.147 |
| 84 | 0.13 |
| 96 | 0.10 |

**Table S4** Effect of metal ions and modulators on glycosylated Ba-Gt-amy

| **Metal ions (1 mM)** | **Ba-Gt-amy*** | **Ba-Gt-amy**** |
| --- | --- | --- |
| Control | 100 | 100 |
| Co^2+^ | 215 ± 2.4 | 211 ± 2.0 |
| Pb^2+^ | 72 ± 1.3 | 66 ± 1.5 |
| K^+^ | 98 ± 2.1 | 97 ± 1.9 |
| Mn^2+^ | 21 ± 1.1 | 24±.1.3 |
| Cu^2+^ | 92 ± 1.1 | 90 ± 1.4 |
| Fe^2+^ | 140 ± 2.5 | 132 ± 1.5 |
| Ca^2+^ | 100 ± 0.9 | 100 ± 1.6 |
| Ag^+^ | 86 ± 1.0 | 80 ± 0.8 |
| Ni^2+^ | 62 ± 1.4 | 58 ± 1.7 |
| Sn^2+^ | 85 ± 2.2 | 80 ± 1.5 |
| Cd^2+^ | 88 ± 1.4 | 85 ± 0.9 |
| Na^+^ | 97 ± 1.5 | 95 ± 1.5 |
| Mg^2+^ | 110 ± 2.5 | 115 ± 2.76 |
| Hg^2+^ | 0 | 0 |
| **Modulators (1mM)** |  |  |
| EDTA | 85 ± 2.2 | 87± 1.5 |
| EGTA | 100 ± 1.9 | 99 ± 1.2 |
| Phenylmethanesulfonylfluoride | 70 ± 1.9 | 72 ± 1.5 |
| N-bromosuccinimide | 0 | 0 |
| Woodward’s reagent K | 32 ± 1.5 | 38 ± 2.5 |
| β-Mercaptoethanol | 80 ± 2 | 89 ± 2 |
| Dithiothreitol | 90 ± 1.3 | 98 ± 2.3 |
| **Detergents (0.1 %)** |  |  |
| SDS | 95 ± 1.6 | 91 ± 2.4 |
| Tween 80 | 99 ± 1 | 98 ± 1.3 |
| Triton X 100 | 92 ± 3 | 90 ± 3.2 |
| **Organic solvents (10 %)** |  |  |
| Acetone | 115 ± 2.5 | 110 ± 3.5 |
| Ethanol | 111 ± 2.1 | 108 ± 4 |
| Butanol | 106 ± 2.2 | 115 ± 3.2 |
| Isoamylalcohal | 110 ± 1.1 | 112 ± 1.5 |

Results are an average of three separate experiments, with ± indicating standard deviation (SD) about the mean of the triplicates.*from *E. coli; *** from *P. pastoris*

**Table S5** Hydrolysis of soluble and raw starches by Ba-Gt-amy expressed in *P. pastoris*

| Starches | Soluble Potato starch (Sigma) | Buckwheat  starch | Wheat starch | Rice starch | Tapioca | Corn starch |
| --- | --- | --- | --- | --- | --- | --- |
| Ba-Gt-amy* | 48±2.12% | 28.1 ± 2.21% | 32±2.12% | 25±3.5% | 25±2.5% | 28±1.5% |
| Ba-Gt-amy** | 47±2.12% | 26.6 ± 1.1% | 31.1± 1.1% | 27±2.5% | 23.2± 2.1% | 29.1± 2.9% |

Results are an average of three separate experiments, with ± indicating standard deviation (SD) about the mean of the triplicates. * Ba-Gt-amy from *E. coli,* ** Ba-Gt-amy from *P. pastoris*
