# Supplementary figures and images for: Production of Chimeric Acidic α-Amylase by the Recombinant Pichia pastoris and Its Applications
Source: Front Microbiol. 2017 Mar 22;8:493. doi: 10.3389/fmicb.2017.00493 (PMC5360700; doi:10.3389/fmicb.2017.00493)

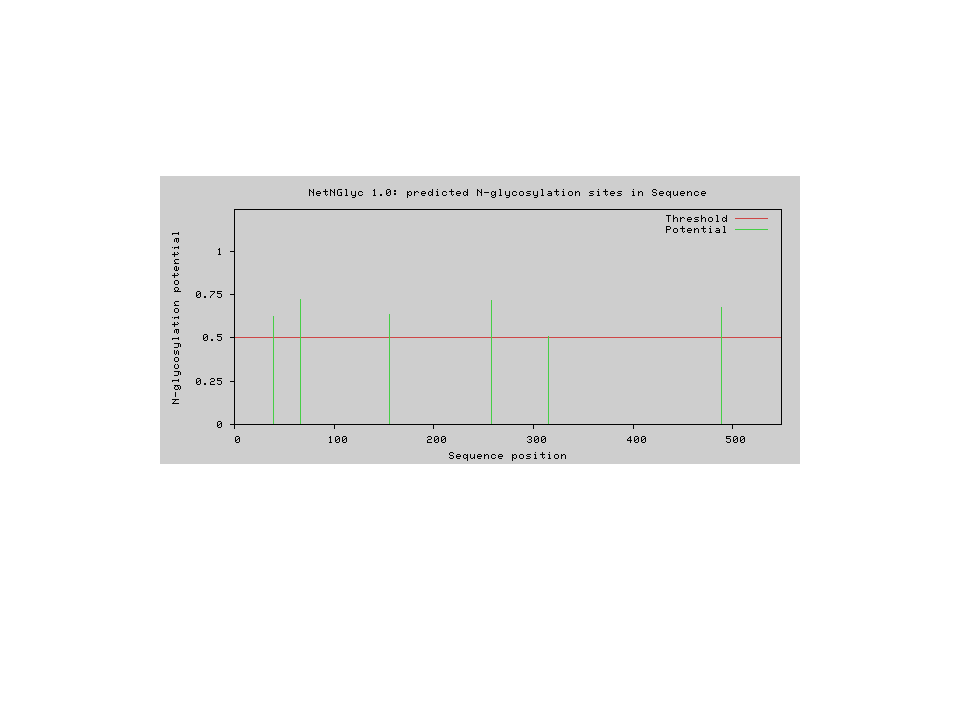

Supplement: Supplementary file 2 [file Image_1.TIF]

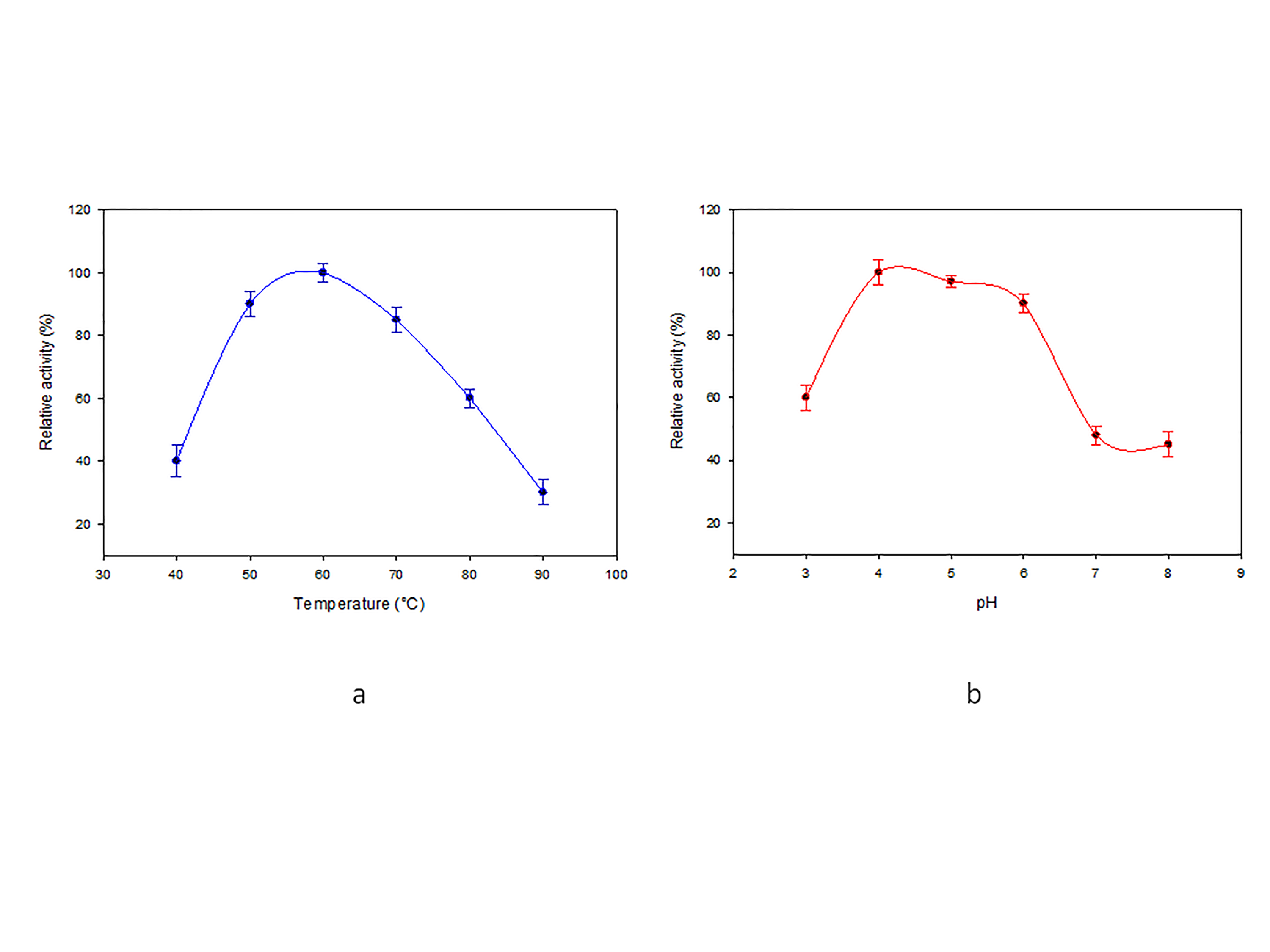

Supplement: Supplementary file 3 [file Image_2.TIF]

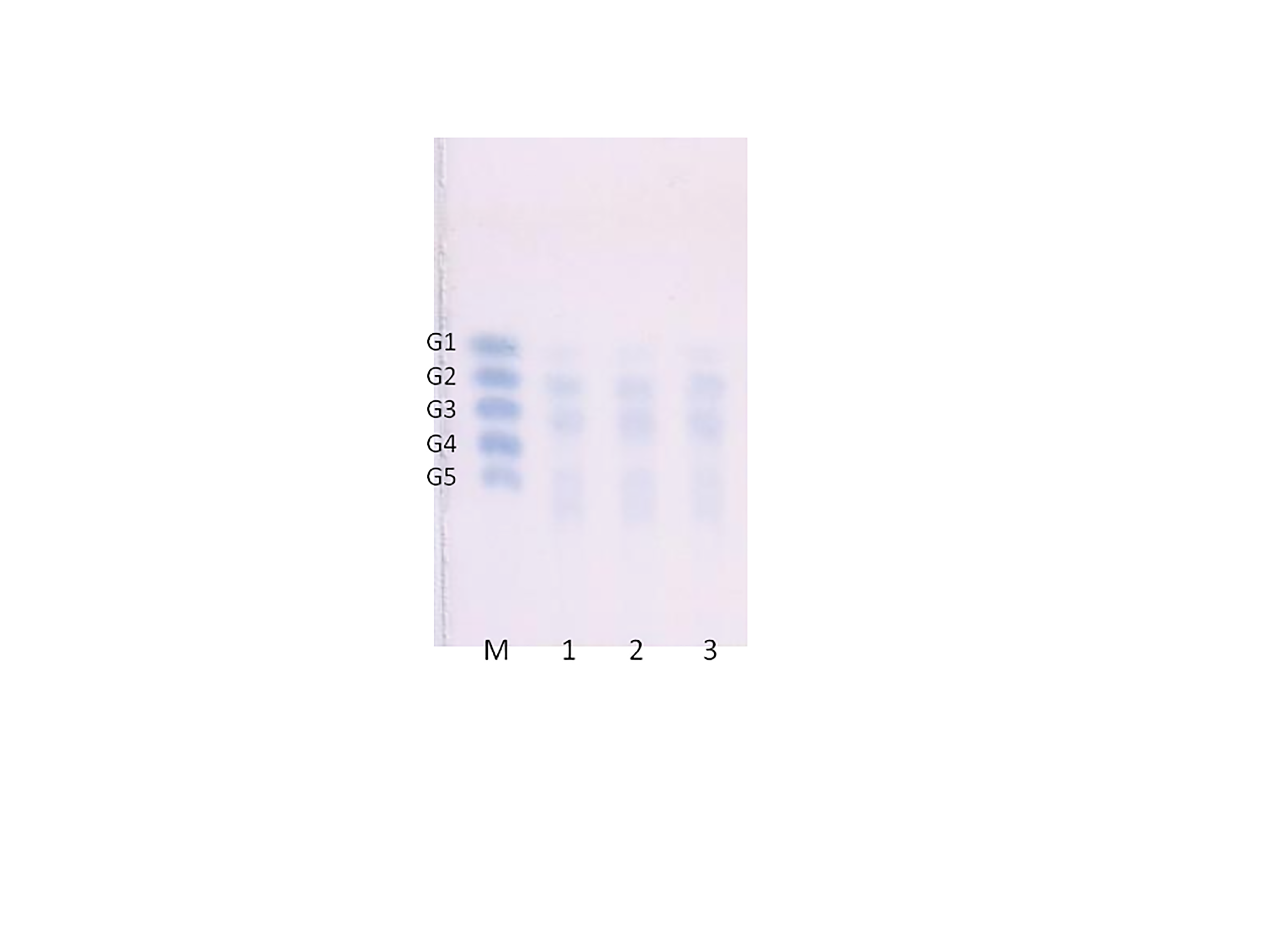

Supplement: Supplementary file 4 [file Image_3.TIF]
